# Label‐Free Dual‐Modal Photoacoustic/Ultrasound Localization Imaging for Studying Acute Kidney Injury

**DOI:** 10.1002/advs.202414306

**Published:** 2025-03-11

**Authors:** Shensheng Zhao, Xingxing Zhang, Keith Bailey, Sathvik Pai, Yang Zhao, Yun‐Sheng Chen

**Affiliations:** ^1^ Department of Electrical and Computer Engineering University of Illinois Urbana‐Champaign Urbana IL 61801 USA; ^2^ Beckman Institute for Advanced Science and Technology University of Illinois Urbana‐Champaign Urbana IL 61801 USA; ^3^ Nick Holonyak Micro and Nanotechnology Laboratory University of Illinois Urbana‐Champaign Urbana IL 61801 USA; ^4^ Department of Psychology University of Illinois Urbana‐Champaign Urbana IL 61801 USA; ^5^ Alnylam Pharmaceuticals Cambridge MA 02142 USA; ^6^ Department of Bioengineering University of Illinois Urbana‐Champaign Urbana IL 61801 USA; ^7^ Department of Biomedical and Translational Sciences Carle Illinois College of Medicine University of Illinois Urbana‐Champaign Urbana IL 61801 USA; ^8^ Cancer Center at Illinois University of Illinois Urbana‐Champaign Urbana IL 61801 USA

**Keywords:** acute kidney injury, label‐free imaging, microvessel imaging, photoacoustic imaging, super‐resolution ultrasound imaging

## Abstract

Growing evidence suggests a close link between acute kidney injury (AKI) and disruptions in renal microcirculation. However, current non‐invasive tools for quantitatively monitoring structural and functional changes in renal microcirculation remain limited, making early diagnosis difficult. To address this challenge, this work introduces a label‐free 3D multi‐parametric imaging technique that combines photoacoustic and super‐resolution ultrasound imaging. This approach provides high‐resolution information on renal vasculature, hemodynamics, and oxygenation. This system offers the ability to visualize the entire renal vasculature at a resolution of 26 µm. In an AKI model, this work demonstrates a 54% reduction in vascular density, a 14.1% decrease in renal oxygenation, and a 61% decline in relative blood volume (rBV) 3 days post‐surgery. The progression of kidney disease is further confirmed through blood tests and histopathological analysis of the collected kidney tissues. These findings indicate that this 3D renal imaging technique holds substantial potential to advance the understanding of renal physiology and offers a valuable tool for investigating renal injury.

## Graphical abstract

This work develops a 3D label‐free photoacoustic/super‐resolution ultrasound imaging system as a comprehensive non‐invasive tool for acute kidney injury (AKI) study. This technique visualizes the intricate structure of renal microvessels and provides critical physiological insights, including renal hemodynamics and oxygenation. With comprehensive high‐resolutionmulti‐parametric sensing, it offers strong potential for investigating renal microcirculation in AKI, enabling earlier detection and improved diagnosis.

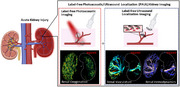

## 1 Introduction

Renal injury, even at relatively mild levels, constitutes a health burden. Recent studies in basic science and epidemiology indicate a causal relationship between acute kidney injury (AKI) and the development and progression of chronic kidney disease.^[^ ^1^, ^2^, ^3^ ^]^ Clinically, AKI may lead to renal vascular occlusion and obstruction and may even impact renal transplantations. AKI is often challenging to completely resolve in many instances, exerting a significant impact on mortality rates and, in certain cases, progressing to terminal renal disease.

Currently, the diagnosis of AKI relies not only on the measurement of acute injury biomarkers but also on steady‐state renal function markers.^[^ ^4^, ^5^ ^]^ However, these methods often suffer from several drawbacks, including retrospection, insensitivity, and potential inaccuracy. Retrospectivity arises because these markers require hours or even days to accumulate. The lack of sensitivity means that low‐level changes are often missed, and since these markers may reflect overall patient conditions rather than specific kidney injury or disease, they can be misleading. These limitations underscore the need for novel approaches to specifically identify and monitor AKI as it progresses.^[^ ^6^, ^7^ ^]^

Increasing evidence suggests that AKI is closely linked to microvasculature dysfunction.^[^ ^8^, ^9^, ^10^, ^11^, ^12^, ^13^ ^]^ This dysfunction leads to reduced renal blood flow, which induces hypoxia, inflammation, and structural changes. However, the tools currently available for non‐invasive and quantitative monitoring of these changes are limited, making early diagnosis challenging. For instance, MRI has been used to monitor vasculature and tissue oxygenation changes in AKI studies,^[^ ^14^, ^15^, ^16^ ^]^ but the need for contrast agents and the insufficient resolution for visualizing microvasculature are significant drawbacks. Preclinical studies using microscopy techniques to image renal microvasculature and tissue oxygenation either have shallow imaging depth or are invasive.^[^ ^17^, ^18^, ^19^ ^]^ Photoacoustic (PA) tomography, while effective in tracking tissue oxygenation and molecular information in deep tissue,^[^ ^20^, ^21^, ^22^, ^23^, ^24^ ^]^ struggles with precise imaging of microvasculature, especially with a linear array setup.

Recently, ultrasound localization (UL) (or super‐resolution ultrasound) imaging has emerged as a promising technique for visualizing microvasculature in deep tissues.^[^ ^25^, ^26^, ^27^ ^]^ It surpasses the ultrasound diffraction limit, enabling us to generate images of vascular structures and flow velocities in deep tissue at a micrometer spatial resolution using FDA‐approved microbubbles as contrast agents. This method has been successfully used to study AKI, revealing significant decreases in microvasculature density.^[^ ^28^, ^29^ ^]^ Furthermore, when combined with PA imaging, it can reveal tissue oxygenation and other molecular information through multi‐wavelength sensing, enabling comprehensive multi‐parametric assessment of the kidney.^[^ ^30^ ^]^ However, the need for continuous contrast agent injections during lengthy data acquisition periods makes these techniques impractical for longitudinal studies that track renal structural and physiological changes over time during AKI.

Label‐free UL imaging offers the potential to overcome these challenges without relying on microbubbles. Techniques such as Doppler slicing^[^ ^31^ ^]^ and deep learning^[^ ^32^ ^]^ have been used to reconstruct super‐resolved vasculature maps from Doppler signals. However, these contrast‐free methods only can visualize vascular structures and have limitations in tracking scatterers to detecting detailed hemodynamic changes, such as blood flow information in vivo. Another approach involves detecting and localizing red blood cells (RBCs) or erythrocytes for super‐resolution imaging,^[^ ^33^ ^]^ which has been validated in rat kidney imaging,^[^ ^34^ ^]^ has not yet been applied to the study of AKI.

Despite the ability of label‐free UL imaging to visualize microvasculature and hemodynamic changes, it lacks the capacity to assess tissue oxygenation, an important biomarker for renal function, making it insufficient for a comprehensive assessment of AKI. To address this limitation, in this paper, we present a 3D label‐free dual‐modal PAUL imaging method with a linear array transducer designed to visualize renal anatomy, hemodynamics, and oxygenation. This approach integrates PA imaging with label‐free UL imaging based on erythrocyte localization into a dual‐modality system, combining the strengths of both techniques. We demonstrate that this label‐free imaging system can visualize the entire renal vasculature with a resolution of 26 µm. We further validate this imaging technique in an AKI model, revealing a 54% decrease in vasculature density, a 14.1% decrease in renal oxygenation, and a 61% decrease in relative blood volume (rBV) after 3 days of the surgery. The development and progression of kidney disease were confirmed through in vitro blood tests and histopathological analysis of the collected kidneys. Our findings suggest that this 3D renal imaging technique holds substantial potential not only for advancing our understanding of renal physiology but also as a valuable tool for the early detection and monitoring of kidney diseases and evaluation of treatment outcomes.

## 2 Results

### 2.1 3D Label‐Free PAUL Imaging System for Renal Imaging

**Figure 1:**
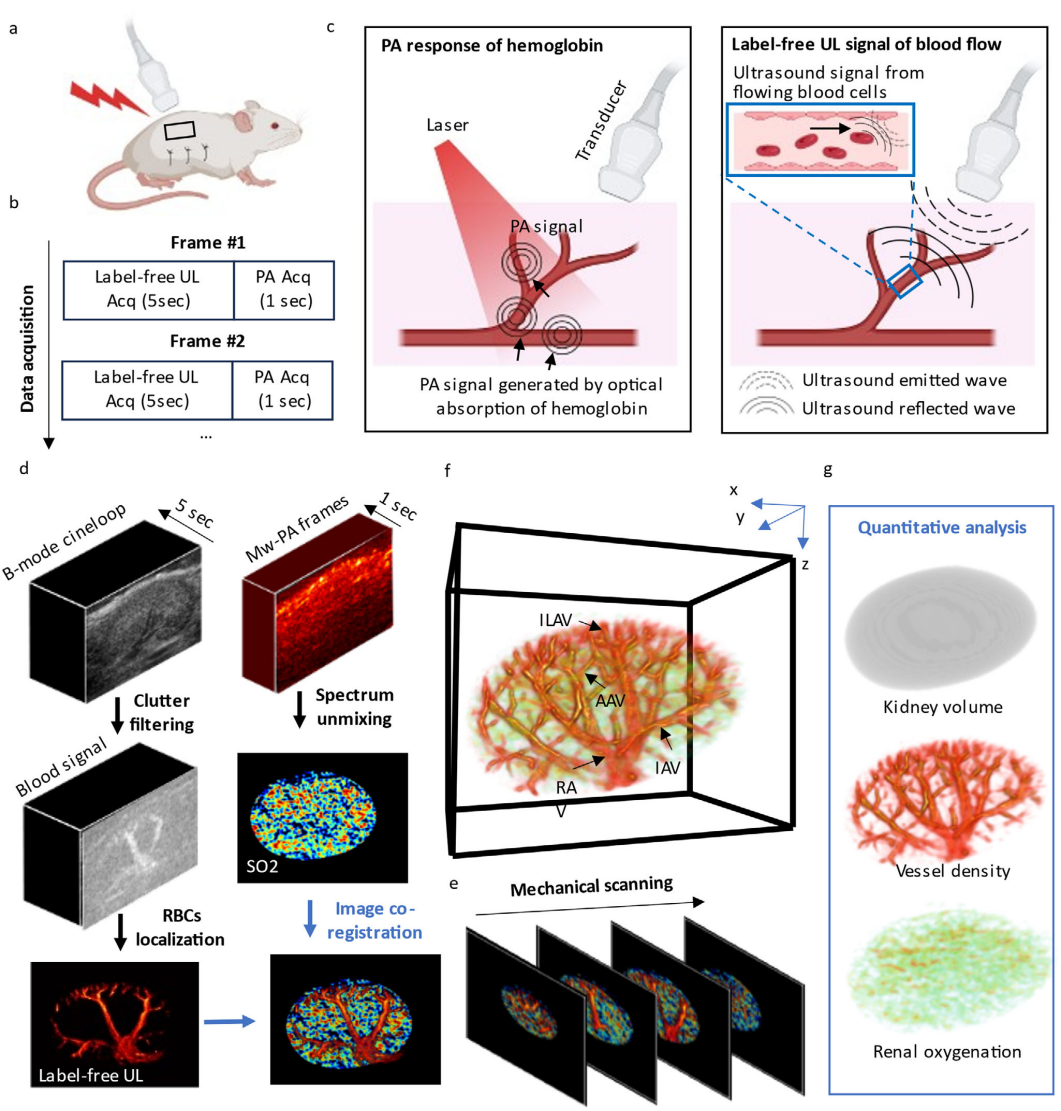
Schematic representation of label‐free 3D PAUL imaging. a) Illustration of kidney imaging with mechanical‐scanning based dual modal photoacoustic (PA) and ultrasound (US) imaging. The central frequency of the diagnostic transducer is 15 MHz. The laser fluence is under 20 mJ cm^−2^. b) During data acquisition, ultrafast ultrasound imaging and multi‐wavelength PA imaging are recorded alternatively at each position. For each position, US images with 5 s acquisition were collected at frame rate of 500 Hz. Multiwavelength PA images (MvPA, 750 and 850 nm) with 1 s acquisition were collected at frame rate of 10 Hz. c) Schematic of PA and label‐free UL acquisition. d) Flowchart of dual modal photoacoustic and ultrasound imaging postprocessing. Clutter filtering was applied to extract blood signal and label‐free super‐resolution ultrasound image was generated by localizing erythrocytes within blood signals. Functional photoacoustic information was extracted by spectrum unmixing of MwPA images. e) Mechanical scanning was conduct to collect 3D kidney structural and physiological information. f) One representative whole kidney microvasculature and oxygenation map of a mouse. g) Quantitative analysis of kidney volume, kidney vasculature and kidney oxygenation from 3D dual modal photoacoustic and ultrasound image. ILAV, interlobular artery‐vein; RAV, renal artery‐vein; IAV, interlobar artery‐vein; and AAV, arcuate artery‐vein.

**Figure** **1**a shows the imaging scheme of 3D label‐free PAUL imaging. A Verasonics imaging system was used to collect PA and ultrasound signals through a 15 MHz customized linear array transducer combined with a customized bifurcated fiber bundle. The 3D data was collected by mechanical scanning of the linear array to collect data frame by frame. In each position, the interleaved imaging sequences were designed to acquire both PA and ultrasound images (Figure 1b). The contrast of PA imaging in this study is mainly from hemoglobin (Figure 1c). For each photoacoustic acquisition, after laser activation, the transducer detects ultrasound waves generated by hemoglobin following optical absorption. On the other hand, label‐free UL imaging is based on the movement of RBCs. For label‐free ultrasound acquisition, we capture ultrasound signals over time, which includes both flowing RBCs and tissue signals (Figure 1c).

Figure 1d,e shows the processing pipeline of the dual imaging system. The ultrasound signal was acquired first, with a frame rate of 500 Hz, and the total data acquisition time was 5 s.

After that, multiwavelength PA images (750 and 850 nm) were collected with 1 s of data acquisition. After image reconstruction, a linear spectrum unmixing algorithm^[^ ^35^ ^]^ was applied to the multiwavelength PA images to differentiate between deoxy‐hemoglobin and oxy‐hemoglobin information and extract tissue oxygenation information. At the same time, spatiotemporal clutter filtering^[^ ^36^ ^]^ was applied to extract blood signals from B‐mode cineloop. To mitigate motion artifacts before clutter filtering, we analyzed the correlation coefficients of the ultrasound frames (Figure S2, Supporting Information). Respiratory phases, which introduce prominent motion artifacts, were easily identified by their low correlation coefficients. Frames with correlation coefficients below the threshold of 0.95 were discarded to exclude those affected by respiratory motion. The remaining frames were then grouped for clutter filtering. Then, the PSF cross‐correlation‐based localization algorithm^[^ ^26^, ^37^, ^38^ ^]^ was applied to localize erythrocytes from blood signals frame by frame, and localized positions were accumulated to generate the final super‐resolution ultrasound renal vasculature map. Figure S3, Supporting Information shows 2D super‐resolution images with and without motion compensation. After applying motion compensation, the vasculature appears sharper and more clearly defined.

Due to the same ultrasound acquisition system, the final renal vasculature map was automatically co‐registered with the tissue oxygenation map. The 3D renal microvasculature with oxygenation was generated by stacking these co‐registered over scanning directions (Figure 1e). The main renal vessels, such as interlobular artery‐vein, renal artery‐vein, interlobar artery‐vein, and arcuate artery‐vein can be identified in the 3D microvascular map. The quantitative information (Figure 1f), such as renal volume, renal vessel density, renal oxygenation, and renal blood flow, can be extracted from Figure 1e.

### 2.2 Characterization of Label‐Free PAUL Imaging System

Instead of localizing injected microbubbles to achieve super‐resolution, in our label‐free imaging, we focus on localizing the RBCs in the bloodstream. With ≈5 million RBCs per cubic millimeter circulating in the body, removing the tissue signal from B‐mode images exposes the speckle pattern generated by the flowing RBCs. By identifying the peaks in this speckle pattern, we can accurately map the vasculature at high resolution, enabling super‐resolution imaging.^[^ ^33^ ^]^

**Figure 2:**
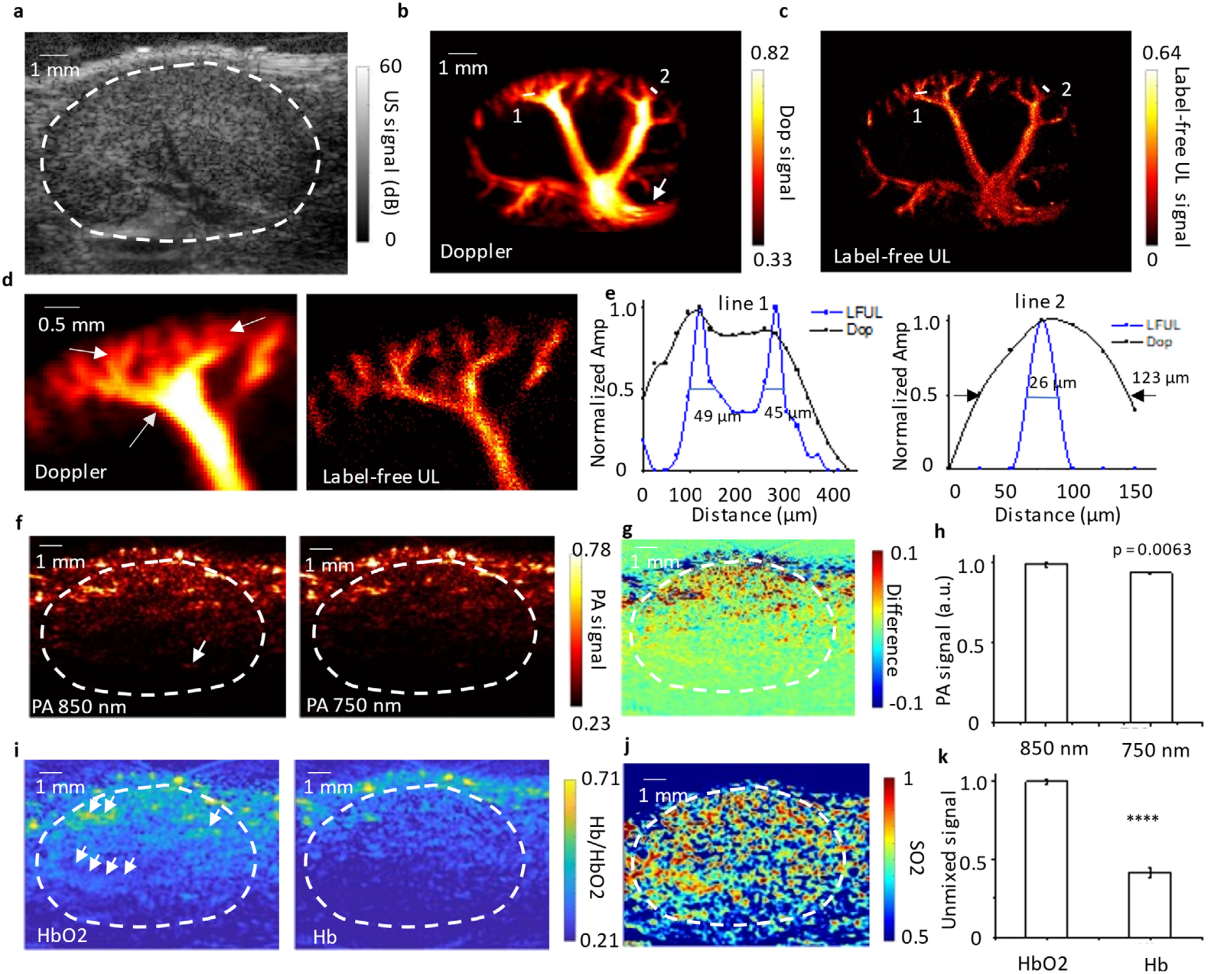
Characterization of label‐free PAUL imaging. a) An ultrasound B‐mode image of a mouse kidney. The boundary of the kidney is indicated by the white dash curve. b) A power Doppler image of a mouse kidney. c) A label‐free super‐resolution ultrasound image of a mouse kidney. (d) Zoom‐in vasculature region from (b) and (c). e) 1D vessel profile of the label‐free UL and power Doppler image at line 1 and line 2 indicated in (b) and (c). f) PA images of a mouse kidney collected at 750 and 850 nm, respectively. g) The difference PA image. h) Comparison of PA signal at 750and 850 nm. The error bars are standard deviations (N = 3). Unpaired student *t*‐test is used. i) Hemoglobin (Hb) and oxyhemoglobin (HbO2) concentration distribution in a mouse kidney. The information was extracted from multiwavelength PA images (750 and 850 nm) via spectrum unmixing. j) Oxygenation saturation (SO2) map of a mouse kidney. k) Comparison of HbO2 and Hb concentration. The error bars are standard deviations (N = 3). Unpaired student *t*‐test is used. ****p < 0.0001.

**Figure** **2**a shows one example of a healthy mouse kidney, where the boundary of the kidney was indicated by the white line. Figure 2b shows mouse power Doppler image of the kidney of the same mouse, and Figure 2c shows the label‐free UL image of the kidney from the same datasets. Compared to the power Doppler image, Figure 2c reveals a more detailed vasculature network. Zoomed‐in images (Figure 2d and Figure S4, Supporting Information) from Figure 2b,c further demonstrate how super‐resolution ultrasound resolves individual vessels, which are obscured by vascular clutters in the Doppler image, as indicated by the white arrows. Quantitatively, we measured two line profiles of vasculatures. Figure 2e shows that label‐free UL image can capture the vasculature as small as 26 µm, whereas the power Doppler can only capture the vasculature at most 123 µm, indicating a 4.7‐fold resolution improvement. To balance scanning time and spatial resolution, we selected a 5‐s data acquisition time for each position. This choice helps minimize the overall mechanical scanning time of the entire kidney and reduces the duration of anesthesia. Additionally, to compare the resolution with and without microbubbles (MBs), we conducted UL imaging of the kidney with and without MB injection in the same position, as shown in Figure S5, Supporting Information. The results indicated that label‐free UL had a lower resolution by an average of 9.03 ± 2.65 µm compared to MB‐UL.

To capture physiological oxygenation information, PA imaging is used here as complimentary imaging. Figure 2f shows PA images of the kidney acquired at 850 and 750 nm illumination, corresponding to the absorption peaks of HbO2 and Hb, respectively, the two main contrasts in PA imaging. Hb is more prominent at 750 nm, while HbO2 is more prominent at 850 nm. Unlike the B‐mode image, a single‐wavelength PA image alone cannot delineate the kidney boundary due to the transducer's limited view. However, with the co‐registered B‐mode image (Figure 2a) and differential PA image (Figure 2g), the kidney boundary can be clearly identified. Additionally, due to the limited‐view of the linear array,^[^ ^39^, ^40^, ^41^, ^42^ ^]^ PA image cannot fully visualize renal vasculature. The difference between the PA images at 850 and 750 nm highlights that the PA signal in the kidney is stronger at 850 nm, whereas in the skin, it is stronger at 750 nm. This indicates that the skin contains more Hb than HbO_2_, while in the kidney, the concentration of HbO_2_ is higher than that of Hb.

Figure S6, Supporting Information shows PA kidney images using a dB‐scale dynamic range which enhances contrast in low signal‐to‐noise ratio (SNR) regions and allows better visualization of PA signals at greater depths. To assess whether deep‐tissue PA signals remain statistically significant for quantitative analysis, we calculated the SNR across the entire kidney. As shown in Figure S7, Supporting Information, the SNR remains above 25 dB and contrast‐to‐noise ratio (CNR) is over 15 dB even at the lower kidney boundary, confirming the system's capability to image the full organ. To further validate the imaging depth of our PA system, we conducted an additional in vivo kidney imaging experiment (Figure S8, Supporting Information). The PA image in Figure S8, Supporting Information reveals deep structures at a maximum observed depth of 10.5 mm. This result aligns with previously reported PA imaging studies using side illumination,^[^ ^42^, ^43^ ^]^ further validating our system's ability to visualize deep tissue (≈10–15 mm at 15 MHz).

To quantitatively compare the signal differences, three regions in the kidney were randomly selected, and the average signal at both wavelengths was calculated. The results (Figure 2h) show that the signal at 850 nm is higher than at 750 nm, though the difference is small (0.9864 at 850 nm vs. 0.933 at 750 nm). This minor difference arises because both 750 and 850 nm wavelengths capture signals from both Hb and HbO_2_. By applying linear spectral unmixing,^[^ ^35^ ^]^ the separate distributions of Hb and HbO_2_ can be extracted, as shown in Figure 2i. It is evident that HbO_2_ concentration is higher than Hb concentration in the kidney region, consistent with previous studies.^[^ ^44^, ^45^ ^]^ The quantitative comparison of HbO_2_ and Hb from the unmixed signals (Figure 2k) demonstrates significantly higher sensitivity than directly comparing PA signals at different wavelengths (Figure 2h). Additionally, with the concentration information of Hb and HbO_2_, one can calculate the tissue oxygenation of the kidney (Figure 2j). The oxygenation map reveals heterogeneous oxygenation distribution in the kidney, corroborating findings from earlier research.^[^ ^45^, ^46^ ^]^

### 2.3 Establishing a Mouse Model of AKI through Renal Ischemia‐Reperfusion Surgery

**Figure 3:**
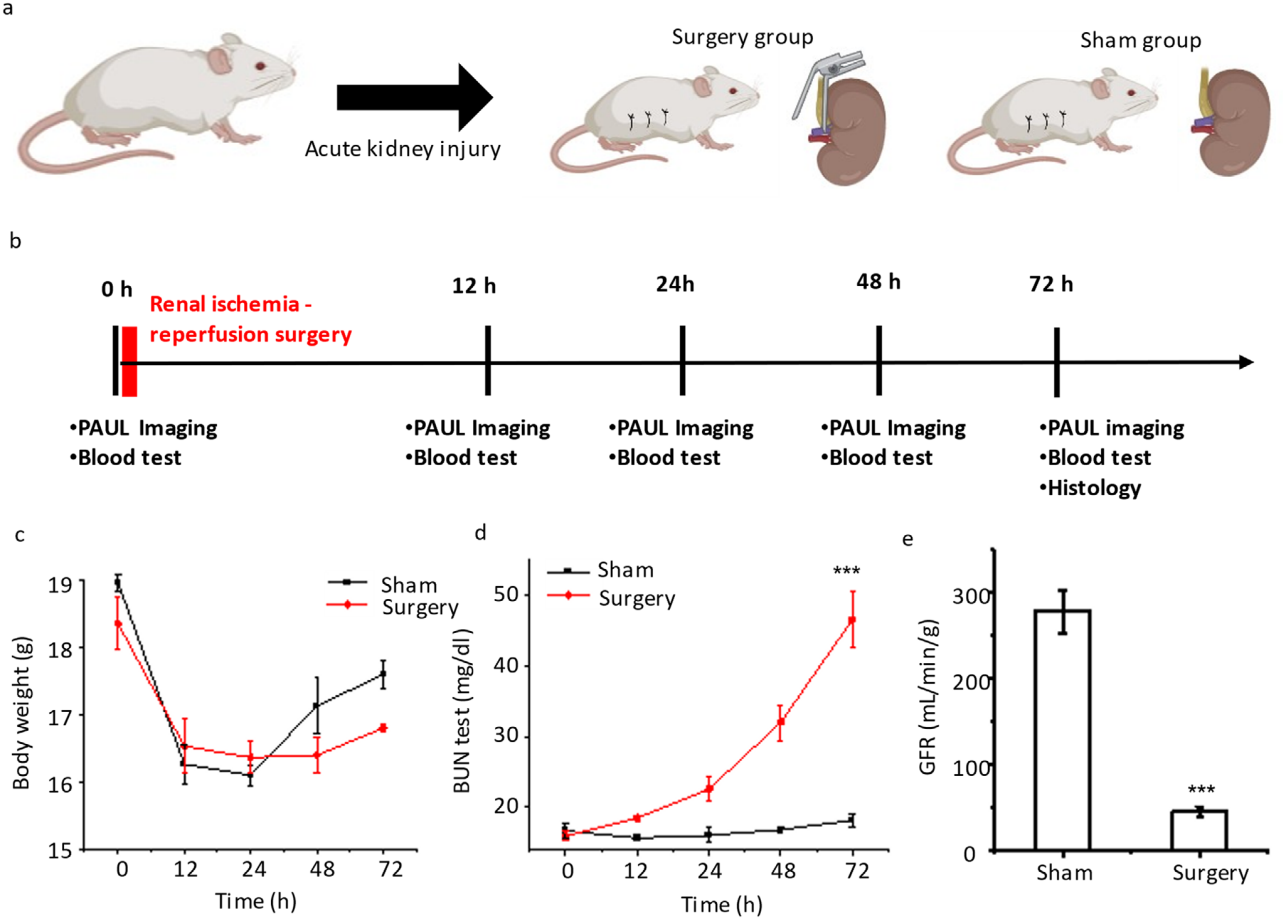
Establishment and Validation of AKI model. a) Illustration of renal ischemia‐reperfusion surgery in mice model. b) The experiment timeline. This study established an acute kidney injury (AKI) model through renal ischemia‐reperfusion surgery in mice at day 0. Blood tests and kidney images with PAUL imaging were collected within 72 h to monitor AKI induction. The kidneys were harvested and prepared for histology analysis at 72 h of the surgery. c) The weights of mice in the surgical group and sham group were at 0, 12, 24, 48, and 72 h. d) Blood urea nitrogen (BUN) level of mice after ischemia‐reperfusion. e) Normalized GFR at 72 h after surgery. The error bars are standard deviations (N  =  3) in (c–e). Unpaired student *t*‐test is used. ***p < 0.001.

To validate the performance of label‐free PAUL imaging, we conducted ischemia‐reperfusion surgery to develop AKI animal models in mice. The detailed surgery protocol of renal ischemia‐reperfusion is described in Methods. Briefly, mice were randomly divided into two groups: surgery (n = 3) and sham (n = 3) (**Figure** **3**a). In both groups, following anesthesia, a midline abdominal incision was made to expose the renal artery and vein of the right kidney. In the surgery group, the kidney pedicles were occluded for 60 min with clamps. After that, the clamps were removed, the kidney was repositioned, and the incision was sutured. The sham group underwent the same procedure but without the occlusion of the kidney pedicles.

The experiment timeline is illustrated in Figure 3b. Prior to surgery, label‐free PAUL imaging was performed to capture the kidney's structural and physiological information, along with a blood test. At 12, 24, 48, and 72 h post‐surgery, both blood tests and PAUL imaging were repeated. After 72 h, the mice were euthanized, and their kidneys were harvested for histological examination.

After ischemia‐reperfusion surgery in mice, both the surgical group and sham group exhibited a noticeable decrease in body weight at 12 h, gradually recovering by 72 h (Figure 3c). Renal function decline was assessed through the increase in blood urea nitrogen (BUN). Before the surgery, the BUN levels of mice in both groups were ≈16 mg dl^−1^. No significant change in BUN levels was observed in the sham group after surgery. In contrast, post‐surgery mice in the surgery group showed a slight increase in BUN at 12 h, a significant elevation from 24 to 48 h, reaching ≈50 mg dl^−1^ by 72 h (Figure 3d), validating the successful establishment of the AKI model.

Furthermore, another important AKI biomarker, the glomerular filtration rate (GFR), was monitored. GFR was by measuring the rate of disappearance of labeled inulin from the blood. GFR‐Vivo 680, a near‐infrared fluorescent inulin‐based probe, was injected via the tail vein of mice, and the blood samples were collected at 0, 30, and 60 min after the injection. The blood samples were imaged with an IVIS system (ex/em = 670/685 nm) to extract the fluorescence signal decay over‐time (Figure S10, Supporting Information). The GFR was extracted by calculating the decay rate from the fluorescence decay curve. The GFR at 72 h post‐surgery is shown in Figure 3e. In comparison to the sham group (24.90 ± 5.62 mL min^−1^ g^−1^), mice in the surgical group (278.46 ± 45.77 mL min^−1^ g^−1^) exhibited a significant reduction of GFR, further indicating the successful development of the AKI model.

### 2.4 Label‐Free Renal PAUL Imaging during AKI

**Figure 4:**
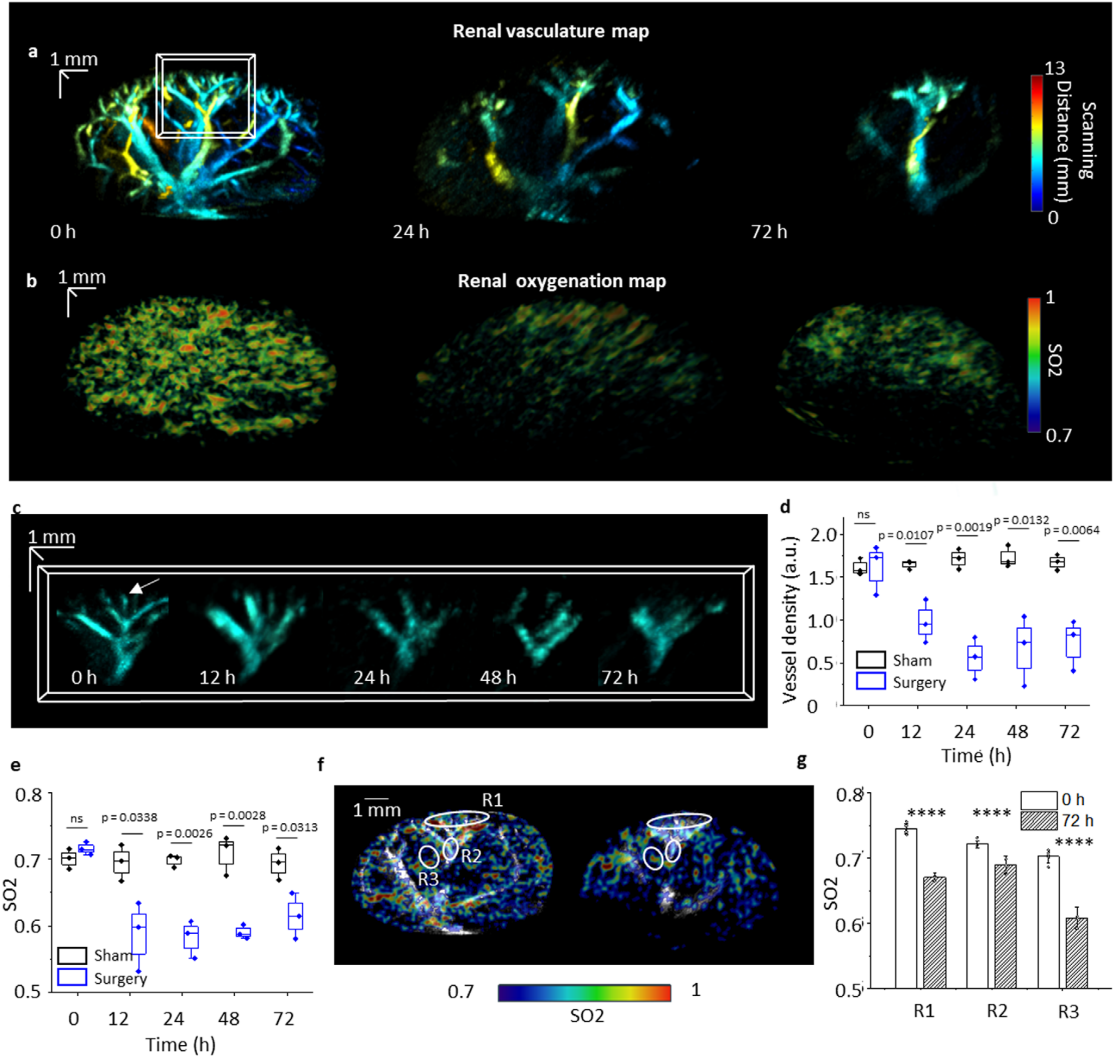
Label‐free PAUL imaging monitors vasculature and oxygenation change in the AKI model. a) Renal vasculature map before, 24, and 72 h of renal ischemia‐reperfusion surgery. The image is rendered with scanning distance color encoded. b) Renal oxygen saturation before, 24 and 72 h of renal ischemia‐reperfusion surgery. c) Zoom‐in renal vasculature before and after 12, 24, 48 and 72 h of the surgery. d) Renal vessel density changes over time in the surgery group and sham group (N = 3). e) Renal oxygenation changes over time in the surgery group and sham group (N = 3). The center line in each box in (d,e) is the median, and the bottom and top edges of the box indicate the 25th and 75th percentiles, respectively. The whisker range is 5th to 95th percentiles. Unpaired student t‐test is used. ns: not significance. f) 2D oxygenation before and after 72 h of the surgery. g) Comparison of oxygenation change in regions indicated in (f) before and after 72 h of the surgery. The error bars are standard deviations (N = 3). Unpaired student t‐test is used. ****p < 0.0001.

To monitor structural and physiological changes during AKI, label‐free PAUL imaging was applied to image the whole kidney of mice in both the surgical group and sham group before and 12, 24, 48, and 72 h after the surgery. **Figure** **4**a shows 3D super‐resolution ultrasound kidney vascular images before and after surgery in the surgery group. Figure S12, Supporting Information reveals kidney vasculature change in another mouse with AKI. After surgery, a significant decrease in vessel density can be found in 24 and 72 h. Figure 4c shows zoom‐in regions of renal vessel branches at different times. It is obvious that some microvasculature (such as ILAV indicated by white arrows) decreases after the surgery and does not recover after 72 h of the surgery. To quantitatively analyze the vasculature change, we calculated vessel density by dividing total renal vessel areas over the kidney volume. We segmented kidney boundaries based on B‐mode image frame by frame, to calculate kidney volume. The renal vessel areas were reconstructed by binarizing the 3D vasculature map and summing all non‐zero voxels. Figure 4d shows vessel density changes over time in both surgery and sham groups. Before the surgery, there was no significant difference in vessel density in these groups. However, the vessel density decreases by up to 67% at 24 h in the surgery group after the surgery compared to the sham group. After 72 h of surgery, the vessel density is still lower than the vessel density before surgery (54% decrease). In the sham group, no significant difference was observed for vessel density at the same time as that in the surgery group.

Instead of structural change, PAUL imaging has the capability to capture tissue oxygenation with multiwavelength PA acquisition. Figure 4b shows the renal oxygenation map before, 24 and 72 h after the surgery. Compared to renal oxygenation before surgery, the signal is much lower after surgery. Figure 4e shows quantitative changes in renal oxygenation. The average oxygenation of the whole renal is ≈0.71 before the surgery. After surgery, renal oxygenation has a significant decrease at 12 h (16.6%), indicating hypoxia status. After 72 h of surgery, the oxygenation level is still lower than that before surgery (14.1%). However, the sham group has a similar level of renal oxygenation. This result matches a similar study.^[^ ^19^, ^45^ ^]^ Figure 4f shows 2D slices of renal oxygenation overlaid with vasculatures before and 72 h after surgery enabling the identification of different kidney regions for tissue oxygenation analysis. We selected several small regions from the cortex, artery/vein, and medulla to analyze tissue oxygenation (R1‐3 as indicated in Figure 4f). Since PA imaging has much lower resolution than label‐free UL imaging, it cannot resolve individual vessels. Therefore, we use relatively large ROIs to ensure more accurate oxygenation quantification. The analysis revealed a decrease in oxygenation at 72 h post‐surgery (Figure 4g). However, recovery rates varied between different regions. For example, R2 showed a much smaller decrease in oxygenation compared to R3, highlighting a heterogeneous effect of AKI on tissue oxygenation.

**Figure 5:**
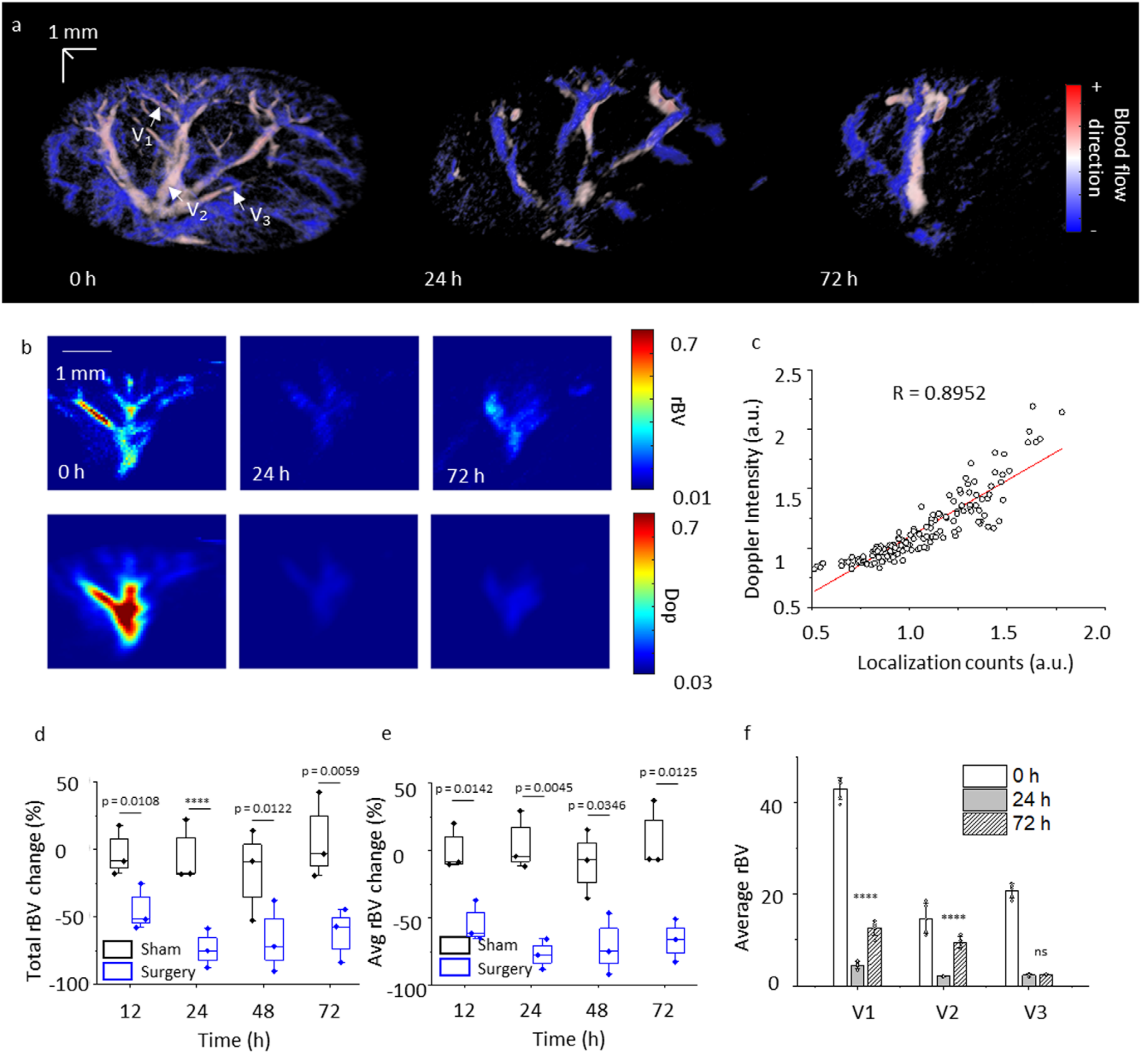
Label‐free PAUL imaging monitors hemodynamics change in the AKI model. a) Renal blood flow direction before, 24 and 72 h of renal ischemia‐reperfusion surgery. b) Zoom‐in rBV maps and power Doppler maps before, 24 and 72 h of renal ischemia‐reperfusion surgery. c) The correlation between power Doppler signal and erythrocytes localization numbers. d) Total relative blood volume (rBV) changes over time in the surgery group and sham group (N = 3). e) Average rBV changes over time in the surgery group and sham group (N = 3). The center line in each box in (d‐e) is the median, and the bottom and top edges of the box indicate the 25th and 75th percentiles, respectively. The whisker range is 5th to 95th percentiles. Unpaired student *t*‐test is used. ****p < 0.0001. f) Comparison of rBV change in regions indicated in (d) before and after 24 and 72 h of the surgery. The error bars are standard deviations (N  =  3) in (c–e). Unpaired student *t‐*test is used. ****p < 0.0001. ns: not significance.

Hemodynamics is another important parameter for AKI. Figure **5**a shows the blood flow direction of the whole kidney at different times of AKI, where flow decreases in both flow directions after the surgery. The flow direction indicates the blood flow toward (+) or away (‐) to the ultrasound imaging transducer. Because hemodynamics is related to the movement of erythrocytes, with the capability of label‐free PAUL imaging to localize erythrocytes, the counts of localization points can indicate hemodynamic change. To validate this assumption, we processed the power Doppler kidney image with the same data and correlated the power Doppler intensity with localization points. It is well known that power Doppler intensity is proportional to rBV,^[^ ^47^ ^]^ an important hemodynamic indicator. Figure 5b compares the Doppler signal and localization signal in a small kidney vessel branch. A decrease in both Doppler and localization signals was observed after the surgery. The signal distribution of Doppler and localization follows a similar trend, indicating a strong correlation between them. Figure 5c shows the correlation between power Doppler intensity and localization points. The high correlation (R = 0.8952) validates localization points can present rBV.

Figure S13, Supporting Information shows a 2D cross‐section of the renal RBC distribution map in the surgery group. Similar to microvascular change, we found that rBV decreases after surgery. Quantitatively, we calculated rBV change over time. Figure 5d,e shows the total rBV change and average rBV change, respectively. The total rBV was calculated by accumulating all localization counts in the whole kidney, while the average rBV was calculated by averaging localization counts in the whole kidney in terms of kidney volume. Both total and average rBV decreases over time in the surgery group, and they reach the minimum (−73.6% for total rBV vs. −76.9% for average rBV) at 24 h after surgery, like the change of vessel density. Then the rBV slightly increases at 72 h, indicating the recovery process of kidney injury. In contrast, the rBV change in the sham group has limited variation (15.7%). The statistical power and Type 2 error for total rBV and average rBV changes at different time points are presented in Tables S1 and S2, Supporting Information. Despite the small sample size (n = 3 per group), the statistical power for average rBV change—calculated by normalizing total rBV with the scanned kidney volume—remains consistently high (>0.8). This indicates a strong likelihood of correctly detecting a true effect. For total rBV change, the statistical power is also generally high (> 0.8) except at 48 h, where it is notably lower. This may be because one sample has incomplete kidney coverage during scanning, causing large variations in the control group's total rBV measurements, which increases the Type 2 error. However, using average rBV by normalizing total rBV with the scanned kidney volume, we reduce variability and improve measurement accuracy.

Leveraging the super‐resolution capability of label‐free PAUL imaging, we can analyze rBV changes at single vessel scale. We selected 3 vessels, as indicated in Figure 5a and Figure S13, Supporting Information, and monitored the average rBV change in these vessels over time in the surgery group. Figure 5g shows the average rBV for these vessels at 0, 24, and 72 h after the surgery. We observed a significant decrease in rBV at 24 h after surgery. Different vessels have different rBV changes at 72 h, indicating a heterogeneous recovery among the vessels. For example, vessels 1 and 2 showed an increase in rBV at 72 h, suggesting a recovery in vessel structure. In contrast, vessel 3 exhibited similar rBV values at both 24 and 72 h.

To further illustrate these findings, we provide 3D visualizations of renal vasculature from a scanning depth of 5 mm to 7.5 mm, where individual vessels are more distinguishable (Figure S14, Supporting Information). These visualizations reveal a marked reduction in vascular density post‐surgery, emphasizing the extent of vascular damage. The ischemia‐reperfusion site was located on the right side of the kidney, as confirmed by B‐mode imaging in Figure S11, Supporting Information. Compared to the unaffected left side, the right kidney exhibited a greater reduction in vessel density at 72 h, particularly in L1, L2, and S4, indicating more severe vascular damage. In contrast, left‐side regions such as S5 and L3 showed signs of vascular recovery.

To quantitatively assess these trends, we analyzed the average signal intensity over time in L2, L3, S4, and S5 (Figure S14, Supporting Information). The results indicate that vessels closer to the reperfusion site (L2, S4) exhibited minimal recovery within 72 h, suggesting greater initial damage and prolonged impairment. Conversely, vessels farther from the reperfusion site (L3, S5) showed early signs of recovery as soon as 24 h post‐surgery, highlighting a spatially dependent healing process.

Additionally, we compared the recovery dynamics of small and large vessels within the same vascular branch. In L3 and S5, both vessel types showed recovery, but small vessels in S5 recovered by ≈75% after 72 h, whereas large vessels in L3 recovered only 48%. This suggests that small vessels recover faster in regenerating regions. However, in areas closer to the reperfusion site, small vessels were more susceptible to initial damage, with a signal decrease of 86% in S4, compared to 73% in L2 after 72 h. These findings indicate a size‐ and location‐dependent recovery process—small vessels are more vulnerable to initial injury near the reperfusion site but tend to regenerate faster than larger vessels in less affected areas. Future studies incorporating histology or molecular imaging could provide further insights into the mechanisms underlying this size‐ and location‐dependent recovery process.

### 2.5 Histology and Inflammation Analysis

**Figure 6:**
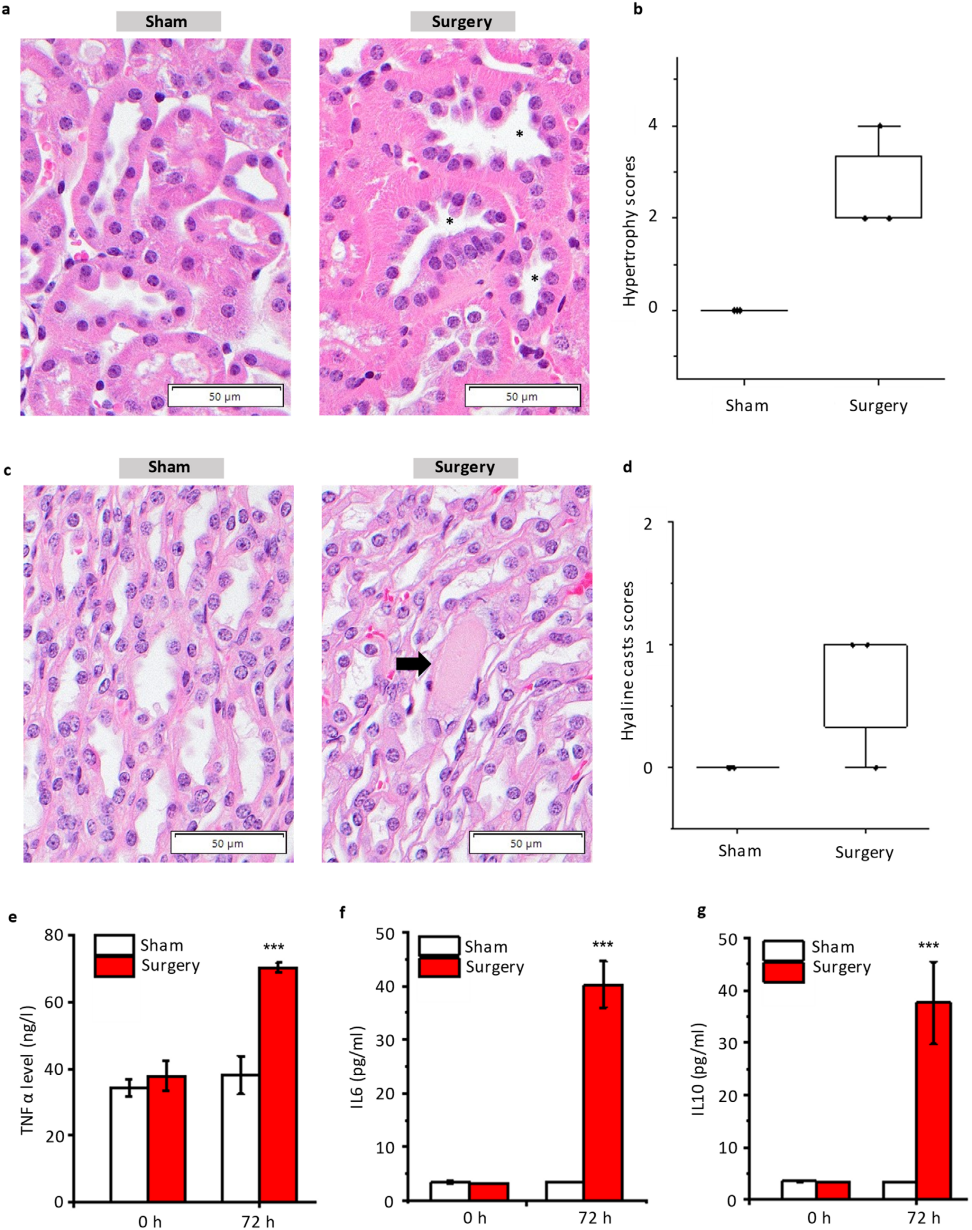
Histology analysis. a) H&E stain images (400×) in surgery group and sham group. Hypertrophy of the distal tubular epithelium (asterisks) was observed for animals in the surgery group and represented a compensatory response to increased renal demand. b) Hypertrophy scores in surgery group and sham group (N = 3). The bottom and top edges of the box indicate the 25th and 75th percentiles, respectively. The whisker range is 5th to 95th percentiles. c) H&E stain images (400×) in surgery group and sham group. Hyaline casts were observed in papillary tubules (arrow) of surgery animals, indicating glomerular damage with resultant increased glomerular permeability and protein leakage. d) Hyaline casts scores in surgery group and sham group (N = 3). The bottom and top edges of the box indicate the 25th and 75th percentiles, respectively. The whisker range is 5th to 95th percentiles. e–g) TNFα, IL‐10, and IL‐6 levels in the blood of sham and surgery mice at 0 and 72 h. The error bars are standard deviations (N  =  3). Unpaired student *t‐*test is used. ***p < 0.001.

We conducted H&E staining on kidney tissue 72 h after surgery to assess structural changes. Figure S15, Supporting Information present representative sections from the surgery and sham groups, with magnified views in **Figure** **6**a,c. The surgery group displayed a more multifocal distribution, suggesting a heightened inflammatory response post‐surgery. This increased inflammation is corroborated by changes in pro‐inflammatory cytokines detected in blood samples (Figure 6e,f).

We measured the levels of pro‐inflammatory cytokines TNF‐α and IL‐6 in blood samples collected before and 72 h after surgery (Figure 6e,f). The sham group showed minimal changes in cytokine levels, while the surgery group experienced significant increases, with TNF‐α showing a 1.8‐fold increase and IL‐6 an eightfold increase. Additionally, the anti‐inflammatory cytokine IL‐10 was also measured (Figure 6g), revealing an eightfold increase in the surgery group, with no significant change in the sham group. The simultaneous rise in both pro‐ and anti‐inflammatory cytokines underscores the robust immune response associated with AKI.

Histological examination further revealed that the surgery group exhibited mild to moderate hypertrophy of the tubular epithelium in the outer cortex (Figure 6a) and the presence of hyaline casts in the papilla—findings absent in the sham group (Figure 6c). Hypertrophy of the distal renal tubular epithelium represents a compensatory response to increased renal demand. This adaptation enhances the transcellular transport capacity of the renal tubules. The presence of hyaline casts in the kidney generally indicates increased glomerular permeability following glomerular damage.^[^ ^48^ ^]^ Based on these histological markers (hyaline casts in the papilla, labeled with arrow in Figure 6c; hypertrophy of tubular epithelium, labeled with asterisks in Figure 6a), experts scored the severity of kidney damage. The final scores (Figure 6b,d) indicate more severe damage in the surgery group over the 72‐h observation period.

## 3 Conclusion

In this study, we developed 3D label‐free dual modal PAUL imaging for studying AKI. The PAUL imaging can visualize the whole renal microvasculature with a resolution as fine as 26 µm without the use of contrast agents. In addition, it reveals renal physiological information, such as tissue oxygenation and rBV. These multiparametric features enable its compressive assessment of AKI in preclinical studies. We established an AKI model in mice through renal ischemia‐reperfusion surgery. The label‐free imaging revealed a decrease in vessel structure density, tissue oxygenation, and rBV across the whole kidney within 72 h of AKI onset. Notably, the decreases in vessel density and rBV were more pronounced than the reduction in tissue oxygenation.

Our findings show that hemodynamic changes align closely with variations in vessel density. However, the relative change in tissue oxygenation is less significant compared to vessel density and rBV change. In addition, PAUL imaging's high resolution allows us to differentiate between various kidney vessels and regions, revealing a heterogeneous response during AKI. This means that recovery in terms of vessel structure, oxygenation, and hemodynamics varies across different regions. Further studies are needed to explore the correlation between regional recovery patterns.

A critical factor in PA imaging is its sensitivity to skin tone, which affects penetration depth and quantitative measurements, such as oxygen saturation.^[^ ^49^, ^50^ ^]^ Addressing these variations is essential for ensuring accurate and reproducible assessments, particularly when comparing different subject populations in future AKI studies. Additionally, while PAUL imaging provides high‐resolution, label‐free visualization of renal microvasculature, several practical limitations must be considered to enhance its preclinical applications.

One key limitation is the need for mechanical scanning to capture the whole kidney information, which takes about 10 min and introduces motion artifacts, limiting real‐time applications. Accelerating PAUL imaging could be achieved through both hardware and software improvements, such as using deep learning for mapping vasculature from sparse data and upgrading to 2D array transducers for 3D imaging. Additionally, increasing the laser repetition rate could help speed up data acquisition.

The anisotropic resolution of the imaging system may lead to analysis errors. Although the imaging provides a super‐resolved microvasculature map, the elevational resolution is limited by the transducer aperture, causing inaccuracies in vessel density analysis. Furthermore, due to the experimental setup, where the kidney was repositioned during surgery, 3D image co‐registration is challenging. To address this, using a 2D array for further studies could be beneficial.

Another limitation is the current lack of 3D blood speed information due to the use of a linear array. Advanced processing techniques could potentially address this issue.^[^ ^51^ ^]^ Additionally, variations in kidney damage due to the surgical procedure and the need for more extensive animal studies are important considerations. Cross‐validation of vessel density changes with histology or micro‐CT is also recommended.

In conclusion, our work demonstrates the feasibility and utility of 3D label‐free PAUL imaging for detailed, multiparametric analysis of AKI. By visualizing microvascular changes and providing insights into tissue oxygenation and rBV without the need for contrast agents, this technique offers a comprehensive assessment of renal function in ischemia‐reperfusion induced AKI. The ability to achieve high‐resolution, multi‐parametric imaging positions label‐free PAUL imaging as a promising tool for studying not only AKI but also broader vascular pathologies in organ systems.

## 4 Experimental Section

### Dual Modal Label‐Free PAUL Imaging System

The dual‐modal PAUL system consisted of a Verasonics ultrasound imaging research system (Vantage 256, Verasonics Inc., Kirkland, WA, USA), a 15 MHz customized linear array Vermon), and a wavelength tunable (690–950 nm) OPO laser source with 7‐ns‐pulse and 10 Hz pulse repletion rate (Phocus Essential, Opotek, Inc., Carlsbad, CA, USA). The laser fluence used in the study was 15 mJ cm^−2^. A customized bifurcated fiber bundle was used to deliver light from the laser source. The fiber bundle was integrated into the transducer using a 3D‐printed adaptor for the side illumination. When performing imaging, the transducer was connected to Verasonics hardware for data acquisition of ultrasound and PA signals. This work used a function generator to synchronize the data acquisition and the laser firing. 3D imaging was performed by mechanical scanning the transducer with a step size of 0.2 mm.

### Super‐Resolution Ultrasound Imaging Processing

The ultrasound images were reconstructed using the Verasonics beamformer, and every 500 frames were grouped into ensembles. To estimate motion, this work analyzed the ensemble data by selecting a reference B‐mode image and applying frame‐to‐frame normalized cross‐correlation (using the *corr2* function in MATLAB) to the series of ultrasound kidney images. Frames with low correlation indices, indicating respiratory motion, were identified and discarded by setting a threshold of 0.95. The remaining frames were then regrouped into new ensembles.

Spatiotemporal filtering was applied to the in‐phase/quadrature (IQ) data of each ensemble to extract the blood signals (Figure S2, Supporting Information). After filtering, point spread function (PSF) localization was used to identify the positions of RBCs. These positions were accumulated to reconstruct the final vasculature image.

From the literature,^[^ ^19^, ^52^, ^53^ ^]^ the blood velocity in small renal vasculature and large artery was ≈4 and 27 mm s^−1^, resulting in an RBC displacement of ≈8 and 54 µm per frame at 500 Hz. Given that this imaging field of view was 10 mm × 12.5 mm, the 500 Hz frame rate of ultrasound imaging provides sufficient temporal resolution to track RBC movement across different vessel sizes.

### PA Imaging Processing

The PA images were reconstructed using Verasonics beamformer. Then, the PA image was corrected with a motion matrix from ultrasound images. Then PA signal was compensated by dividing optical fluence, which was collected from the power meter. The linear spectrum unmixing algorithm was applied to multiwavelength PA images to extract hemoglobin concentration ($C_{HbO_{2}}$) and deoxy‐hemoglobin concentration (*C_HbR_*). The oxygen saturation was calculated by $sO_{2}=\frac{C_{HbO_{2}}}{C_{HbO_{2}}+C_{HbR}}.$

### Animal Studies

All procedures performed on mice at the University of Illinois Urbana‐Champaign in this manuscript were approved by the Institutional Animal Care and Use Committee (IACUC) under protocol #23 025. Female (n = 10) wild‐type BALB/cJ mice from Jackson Laboratory, with an average age of 6–8 weeks and an average weight of 18–20 grams, were used. Animals were kept under standard laboratory conditions (temperature ±20 °C, 12‐h light/12‐h dark) with either 3 or 4 animals per cage and were allowed free access to water and food.

### Renal Ischemia‐Reperfusion Surgery

Six female BALB/cJ mice were randomly assigned to two groups: surgery (n = 3) and sham (n = 3). In the surgery group, renal ischemia‐reperfusion was performed as described in related literature.^[^ ^54^, ^55^, ^56^ ^]^ In brief, mice were anesthetized with isoflurane inhalation (initially 5% isoflurane, followed by 2–2.5% with oxygen for maintenance). A midline abdominal incision was made, and the renal artery and vein of the right kidney were exposed. The kidney pedicles were occluded for 60 min. Macroscopically, ischemia was confirmed by dark purple discoloration of the kidney. Reperfusion was confirmed when the kidney color returned to normal after clamp removal. The incision was closed in two layers with 5/0 sutures. After closure, the animals were subcutaneously injected with 0.5 mL (≈0.02 oz) PBS supplemented with 2.4 µg buprenorphine to address fluid loss and provide analgesia. An additional dose of buprenorphine was administered in the morning after surgery to alleviate discomfort. For the sham group, the procedure was identical except that no clamps were used for occlusion.

### Follow‐Up and Serum Measurements

The animals underwent a 3‐day postoperative follow‐up, unless mortality or euthanasia occurred due to illness‐related manifestations (including fur wrinkling, diminished activity, cold touch, or excessive weight loss), or for specified reasons. Throughout the follow‐up period, the animals underwent daily examinations. Weight measurements were conducted at 12, 24, 48, and 72 h postoperatively. On the 72 h, all surviving animals were euthanized, and post‐mortem examinations of the abdominal cavities were performed to inspect the kidneys.

At 12, 48, and 72 h postoperatively, blood samples (50 µL) were collected in both a sham‐operated control group and a surgery group subjected to renal ischemia‐reperfusion surgery via the tail vein for the quantification of serum urea nitrogen using the Urea Nitrogen (BUN) Colorimetric Detection Kit (Thermo Fisher, USA). Additionally, at 72 h postoperatively, another 50 µL blood sample was collected for the measurement of inflammatory factors. TNFα, IL‐10, and IL‐6 concentrations in the plasma were measured using enzyme‐linked immunosorbent assay (ELISA) kits (Thermo Fisher, USA). The assays were conducted in triplicate for each sample to ensure accuracy and reproducibility. The results were expressed in ng L^−1^ for TNF and pg mL^−1^ for IL‐10 and IL‐6.

### GFR‐Vivo 680 Measurements

Before animal injection, a vial of GFR‐Vivo 680 (PerkinElmer) was carefully reconstituted in 1.0 mL of phosphate‐buffered saline (PBS) under subdued light conditions. Subsequently, 100 µL (2 nM) of GFR‐Vivo 680 per 25 g of body weight was administered via tail vein injection to conscious mice. For mice with body weights differing from 25 g, the dosage was adjusted accordingly (e.g., 92 µL for 23 g body weight or 112 µL for 28 g body weight). Following the GFR‐Vivo 680 injection, blood samples (≈30 µL each) were collected at 0, 30, and 60 min and kept on wet ice throughout the collection process.

A standard curve was created for each measurement by serially diluting a mixture of standard solutions (including plasma probes) to generate a range of concentrations. 15 µL of each sample were carefully transferred into wells of a black 384‐well microplate. Each standard was tested in triplicate, and the average values were used to construct the fluorescence concentration equation using simple linear regression, as outlined in the item brochure.

### Histology

After euthanasia, kidneys were harvested. The kidney tissues were fixed in 10% formalin, embedded in paraffin, sectioned at 4 µm thickness, and stained with hematoxylin and eosin (H&E) for microscopic evaluation. Histological assessment focused on the presence and severity of three main findings: hyaline casts in the papilla, hypertrophy of tubular epithelium in the outer cortex, and interstitial mononuclear cell infiltration.^[^ ^57^, ^58^, ^59^ ^]^ The severity of each finding was scored on a scale from 0 to 5, where 0 indicates no observed finding, and 5 indicates severe pathology. The distribution of the findings was also noted as either focal(F = 1) or multifocal (M = 2). For each tissue section, different aspects of the histological assessment were scored, and the final score was obtained by multiplying the score with the distribution results. These final scores were then compared between the sham group and the surgery group.

### In Vivo Kidney Imaging Protocol

Before imaging, the whole‐body hair of the mouse was removed with clippers and depilatory cream. The mouse was then placed on a head pad and was anesthetized with 2% isoflurane at 2 L min^−1^ of oxygen flow. The whole right kidney was imaged before and 12, 24, 48, and 72 h after the surgery with the scanning step size 0.2 mm. The dual‐modal imaging was acquired as follows: For each imaging sequence, ultrafast ultrasound imaging was applied first through seven‐angle (−6° to 6°) plane‐wave transmission to collect 5 s of data with a pulse repetition frequency of 500 Hz. Then, multi‐wavelength PA imaging was performed to collect 5 s of data, with the data of 750 and 850 nm 1 s, respectively.

### Multi‐Parametric 3D Imaging Analysis

The whole kidney volume was segmented using 3D slicer.^[^ ^60^ ^]^ This segmented volume was then applied to the super‐resolution ultrasound image to extract the renal vasculature. A binary mask of the renal vasculature was created using the MATLAB function *imbinarize*. Vessel density was calculated as the ratio of the vasculature mask volume to the total kidney volume.

To analyze blood flow direction, the blood signal was separated into negative and positive frequency components after clutter filtering, representing blood flow toward and away from the transducer, respectively. Localization algorithms were used to extract these blood signals in different flow directions.

For renal hemodynamic analysis, the renal vasculature binary mask was applied to the erythrocyte localization distribution map to exclude non‐vasculature signals. The remaining signals were summed to calculate the total rBV. The average rBV was then determined by dividing the total rBV by kidney volume.

For renal oxygenation analysis, the kidney volume mask was applied to the oxygen saturation map to extract the renal oxygen saturation map. The average oxygen saturation was calculated by summing the renal oxygen saturation values across the kidney volume.

### Data and Statistical Analysis

MATLAB was used to process signals and images. For the image display, the ultrasound images were shown on a logistic scale, and PA, doppler, and UL images were displayed on a linear scale. The rigid image transformation (imregtform in MATLAB) was applied to a time series of UL images and PA images. The pixel size of ultrasound and PA images was 50 µm. The pixel size of the label‐free UL was 25 µm. 3D volume with distance color encoded was rendered with 3D PHOVIS^[^ ^61^ ^]^ and the rest of the 3D volumes were rendered with Amira 2022.1. Data was plotted using Origin 2020.

Glomerular filtration rate, body weight, ELISA data, and parameters from PAUL imaging were compared using an analysis of variance (two‐way) to determine differences between the groups. The 0.05 level of probability will be used as the criterion for significance.

## Conflict of Interest

The authors declare no conflict of interest.

## Supporting information



Supporting Information

## Data Availability

The data that support the findings of this study are available from the corresponding author upon reasonable request.
