# Supplementary material for: Label‐Free Dual‐Modal Photoacoustic/Ultrasound Localization Imaging for Studying Acute Kidney Injury
Source: Adv Sci (Weinh). 2025 Mar 11;12(22):2414306. doi: 10.1002/advs.202414306 (PMC12165073; doi:10.1002/advs.202414306)
Supplement: Supplementary file 1 — Supporting Information [file ADVS-12-2414306-s001.docx]

Supporting Information

**Label-free dual-modal photoacoustic/ultrasound localization imaging for studying acute kidney injury**

Shensheng Zhao^†^, Xingxing Zhang^†^, Keith Bailey, Sathvik Pai, Yang Zhao and Yun-Sheng Chen*

*^†^* Contribute equally

A. B. C. Shensheng Zhao, B.C. Xingxing Zhang, D. Sathvik Pai, E. Keith Bailey, A. B. C. F. H. Yang Zhao, A. B. C. F. G. H. Yun-Sheng Chen

A. Department of Electrical and Computer Engineering, University of Illinois Urbana-Champaign, Urbana, IL, 61801, USA

B. Beckman Institute for Advanced Science and Technology, University of Illinois Urbana-Champaign, Urbana, IL, 61801, USA

C. Nick Holonyak Micro and Nanotechnology Laboratory, University of Illinois Urbana-Champaign, Urbana, IL, 61801, USA

D. Department of Psychology, University of Illinois Urbana-Champaign, Urbana, IL, 61801, USA

E. Alnylam Pharmaceuticals, Cambridge, MA, 02142, USA

F. Department of Bioengineering, University of Illinois Urbana-Champaign, Urbana, IL, 61801, USA

G. Department of Biomedical and Translational Sciences, Carle Illinois College of Medicine, University of Illinois Urbana-Champaign, Urbana, IL, 61801, USA

H. Cancer Center at Illinois, University of Illinois Urbana-Champaign, Urbana, IL, 61801, USA

E-mail: yunsheng@illinois.edu


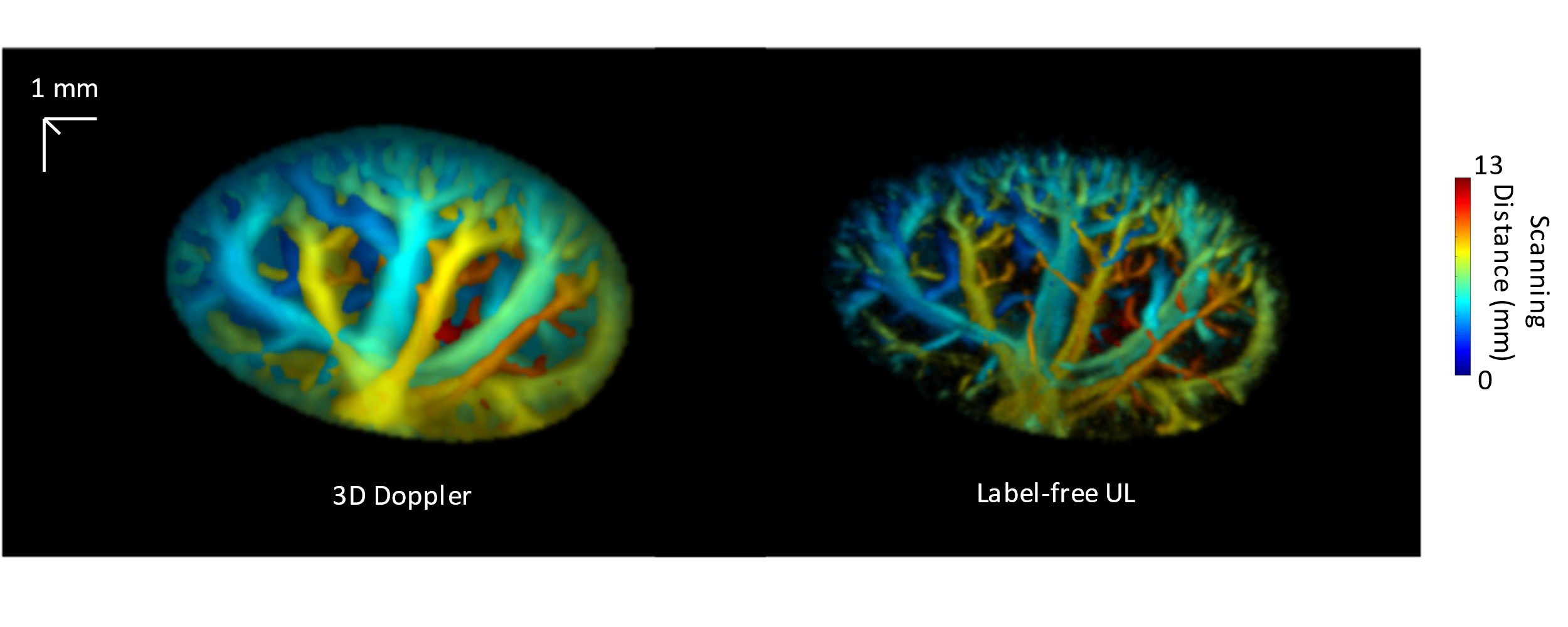


**Figure S1 Comparison of 3D Doppler and 3D label-free UL images in a mouse kidney.**


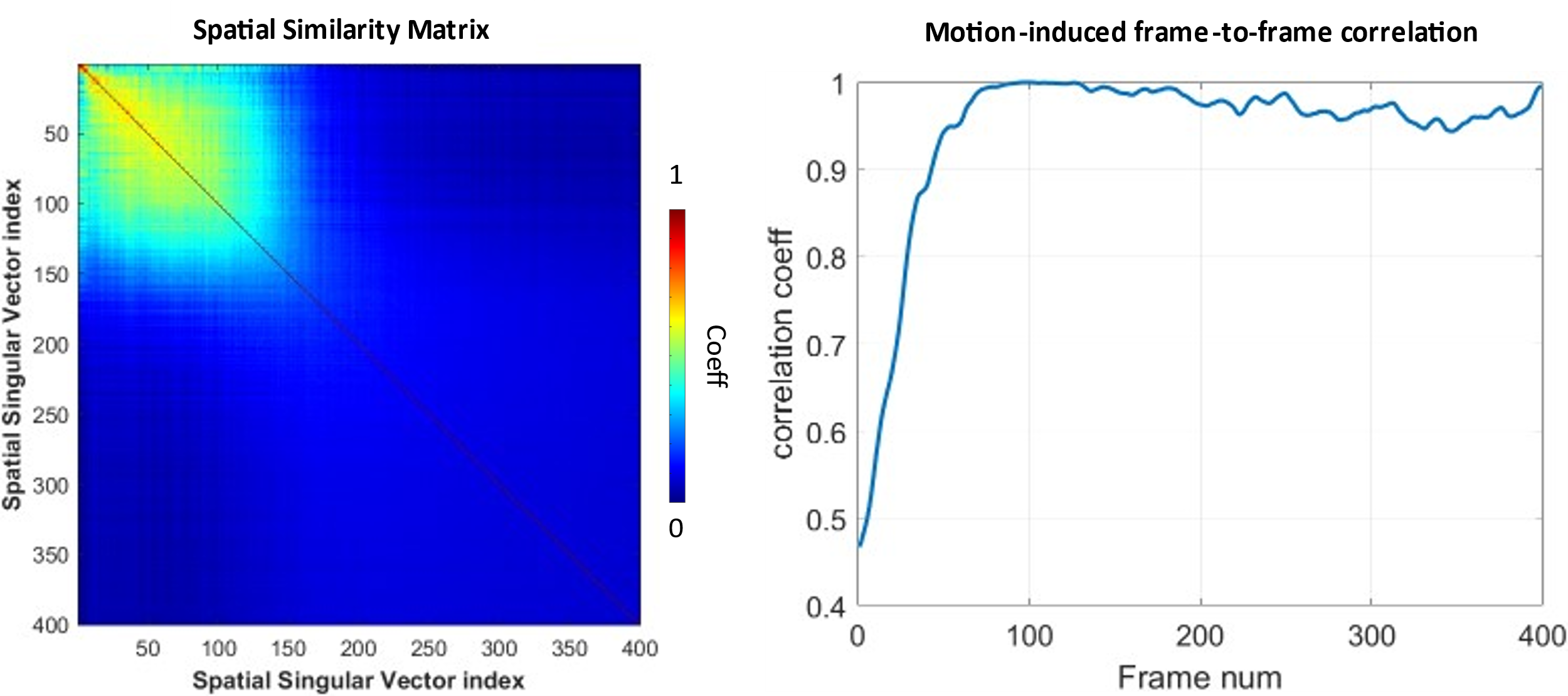


**Figure S2 SVD clutter filtering and motion compensation.**


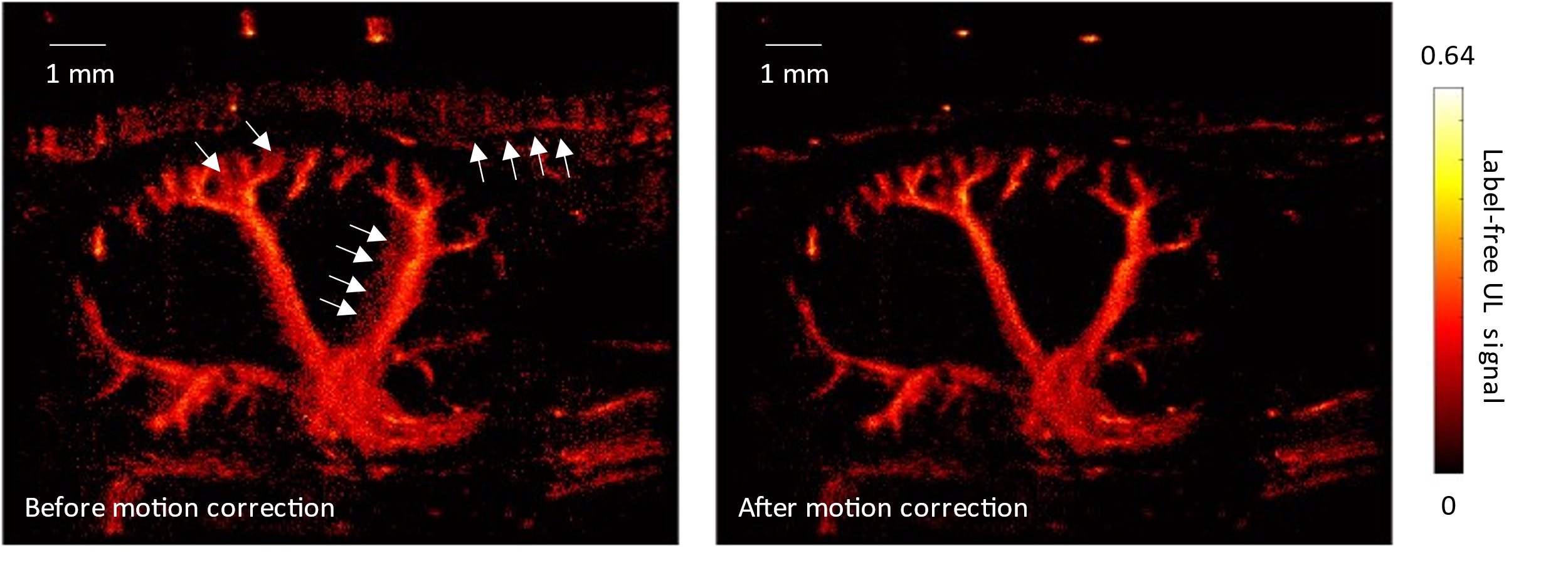


**Figure S3 Lable-free images before and after motion correction.**


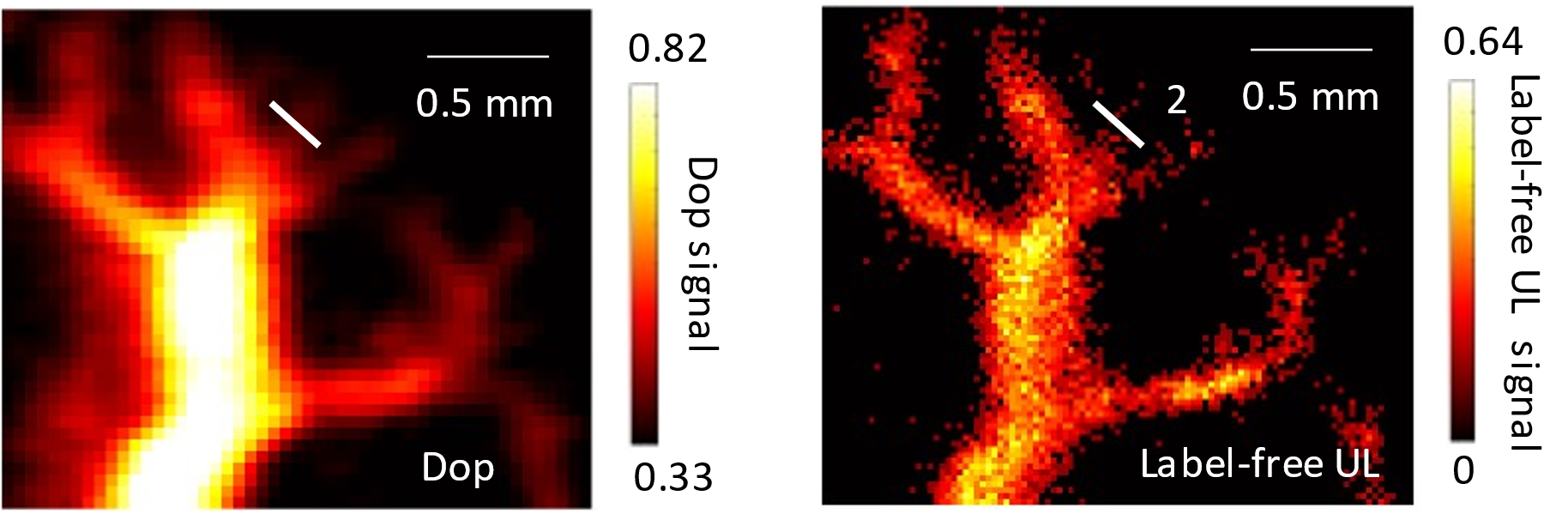


**Figure S4 Zoom-in Doppler and Label-free UL images.**


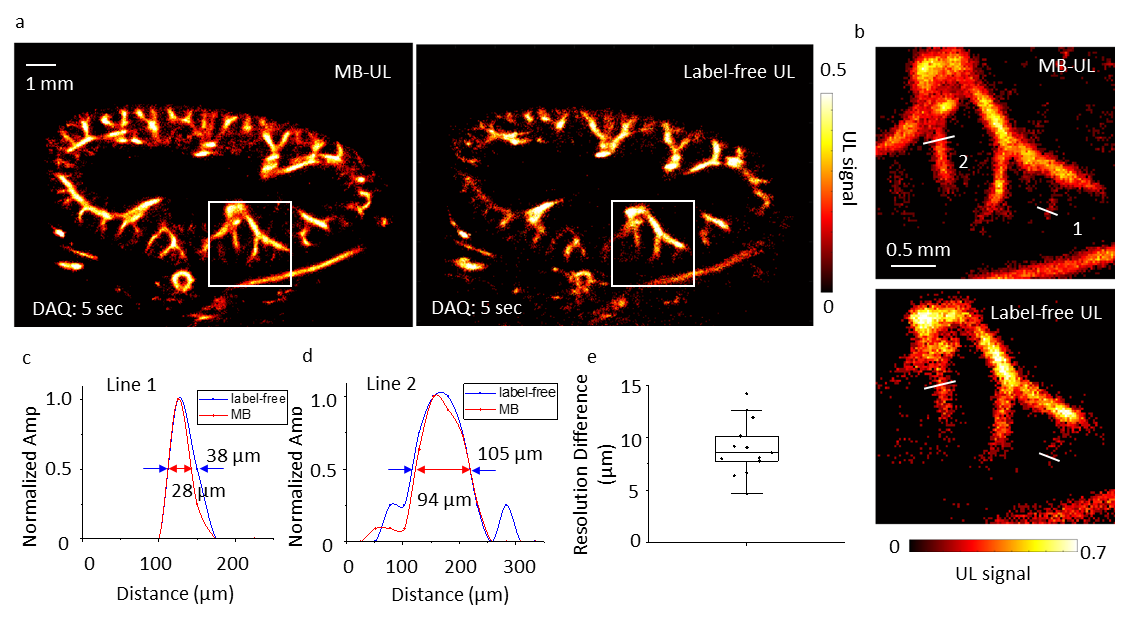


**Figure S5** **Resolution comparison of MB-enhanced UL imaging and label-free UL imaging.** (a) MB-UL and label-free UL image of a mouse kidney with 5 sec DAQ. (b) Zoom-in vasculature images from MB-UL and label-free UL images. (c) The resolution comparison of MB-UL and label-free UL in line 1. (d) The resolution comparison of MB-UL and label-free UL in line 2. (e) The resolution difference between MB-UL and label-free UL images.


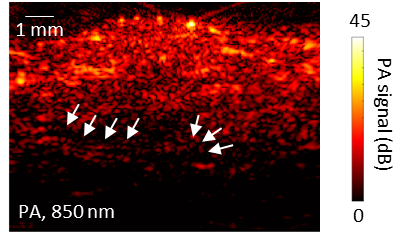


**Figure S6 PA image with dB-scale dynamic range.**


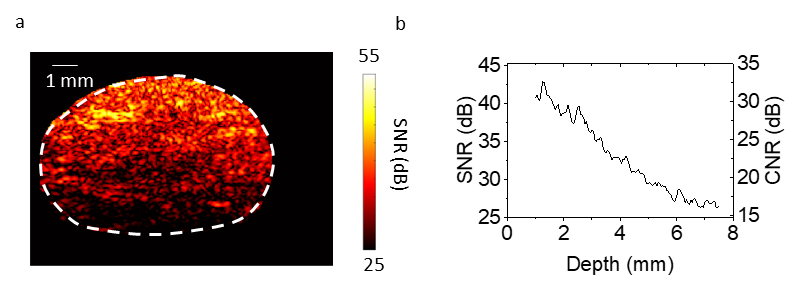


**Figure S7 SNR in photoacoustic kidney imaging.** (a) SNR distribution across the entire kidney cross-section. (b) Average SNR and CNR as a function of imaging depth.


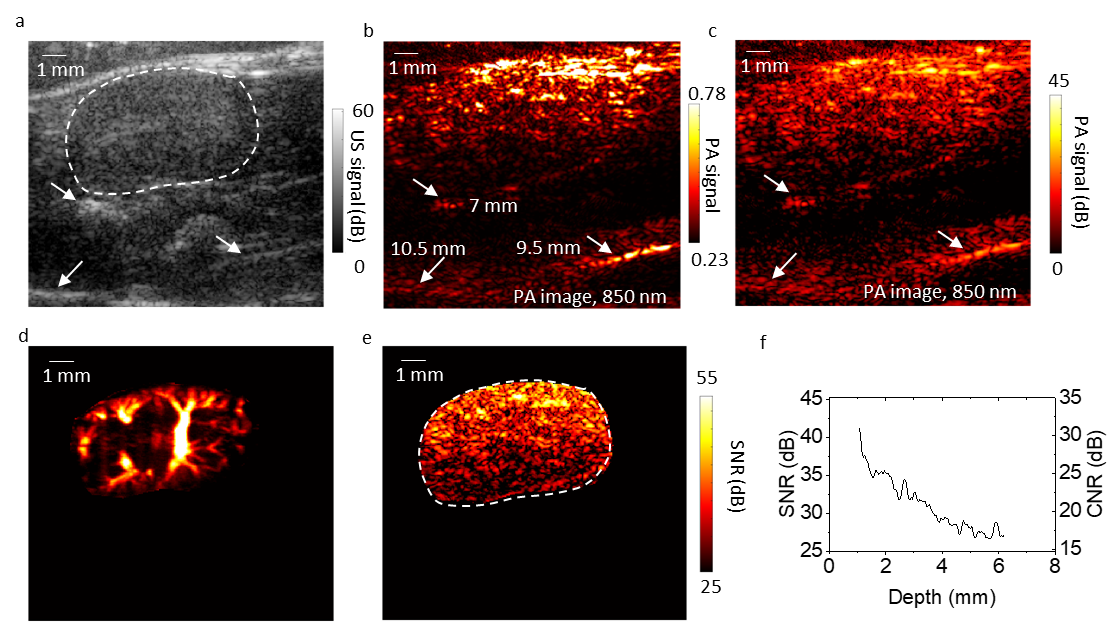


**Figure S8 Imaging depth assessment in PA kidney imaging.** (a) B-mode image of a mouse kidney. (b) PA image of a mouse kidney displayed in a linear dynamic range. (c) PA image of a mouse kidney displayed in a dB dynamic range. (d) Power Doppler image highlighting renal vasculature. (e) SNR distribution of the PA image across the entire kidney cross-section. (f) Average SNR and CNR as a function of imaging depth, illustrating signal variation at different depths.


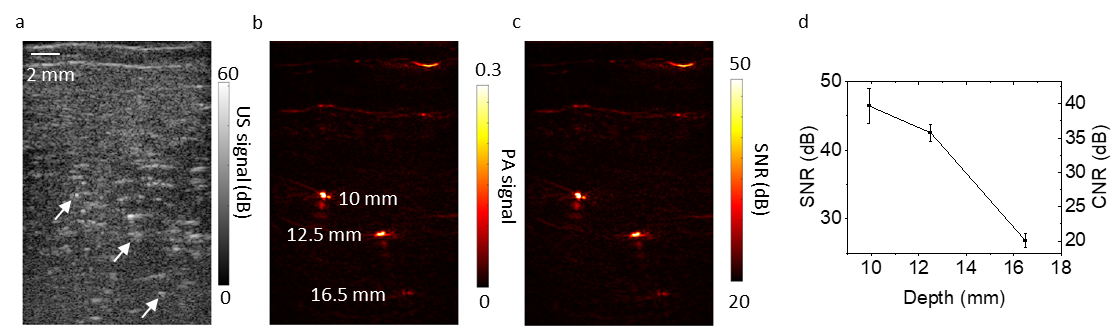


**Figure S9 PA imaging depth in tissue-mimicking phantom.** (a) B-mode image showing a needle inserted into chicken breast tissue, with its position indicated by white arrows.(b) Corresponding PA image of the needle. (c) SNR distribution map of the PA image. (d) Average SNR and CNR of needle targets in photoacoustic image as a function of imaging depth.


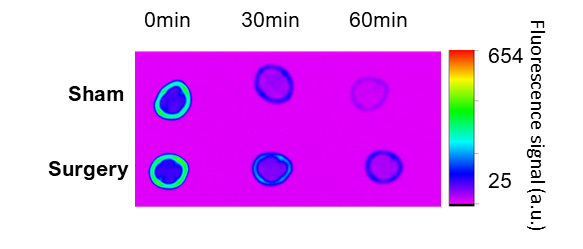


**Figure S10 Fluorescence image of GFR measurement after 72 hours of the surgery in shame and surgery group.**


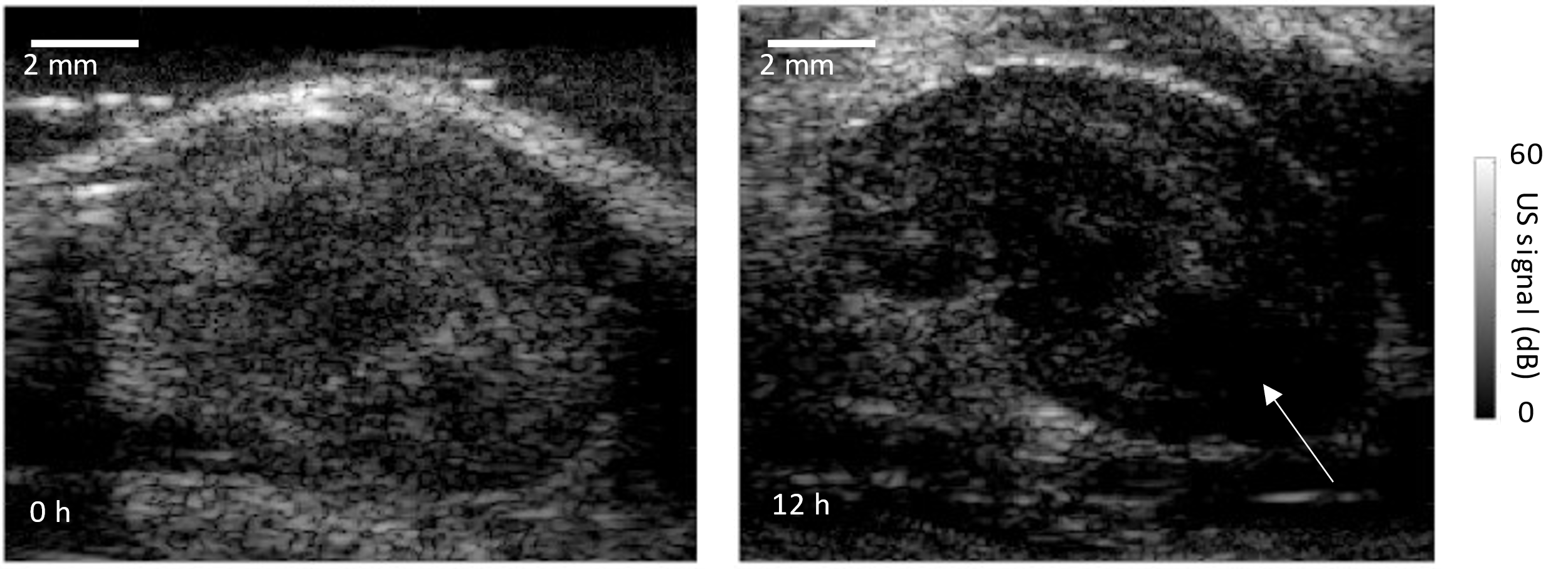


**Figure S11 B-mode kidney images before and 12 hours after the surgery in surgery group.**


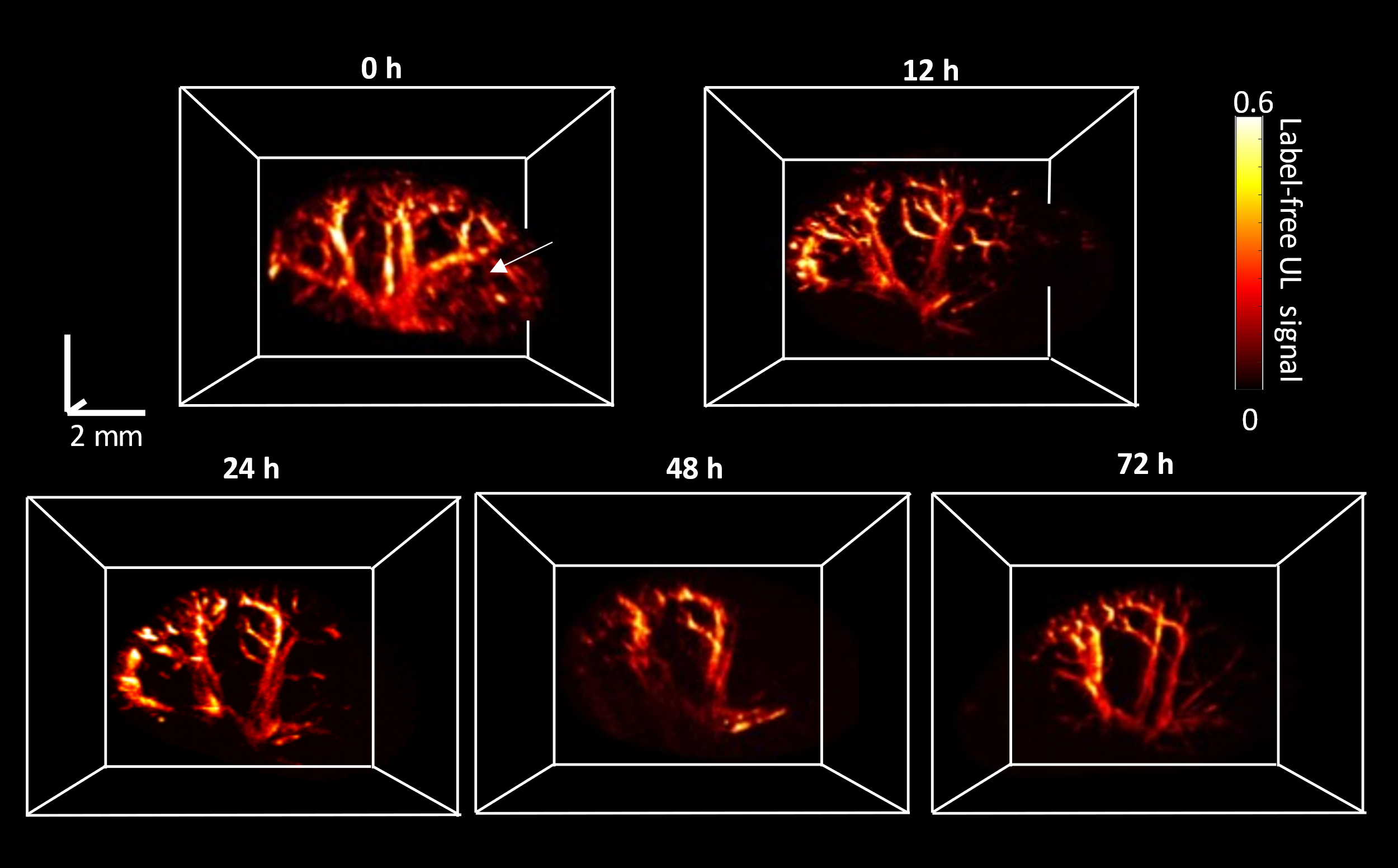


**Figure S12 kidney vasculature change in surgery group.**


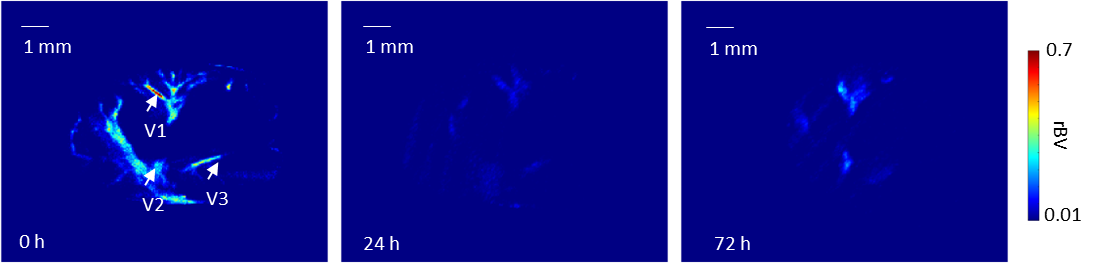


**Figure S13 2D rBV maps before, 24 hours and 72 hours of renal ischemia-reperfusion surgery.**


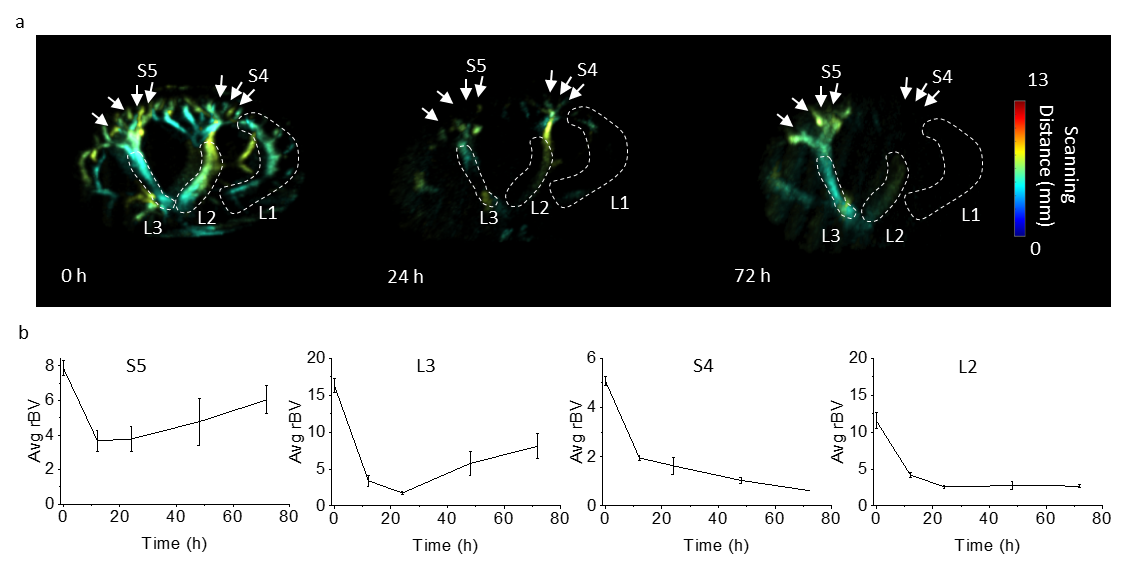


**Figure S14 Quantitative analysis of vasculature changes in AKI.** (a) 3D vasculature maps at baseline, 24 hours, and 72 hours after renal ischemia-reperfusion surgery. (b) Average rBV changes across different vasculatures, highlighting temporal variations in vascular recovery.


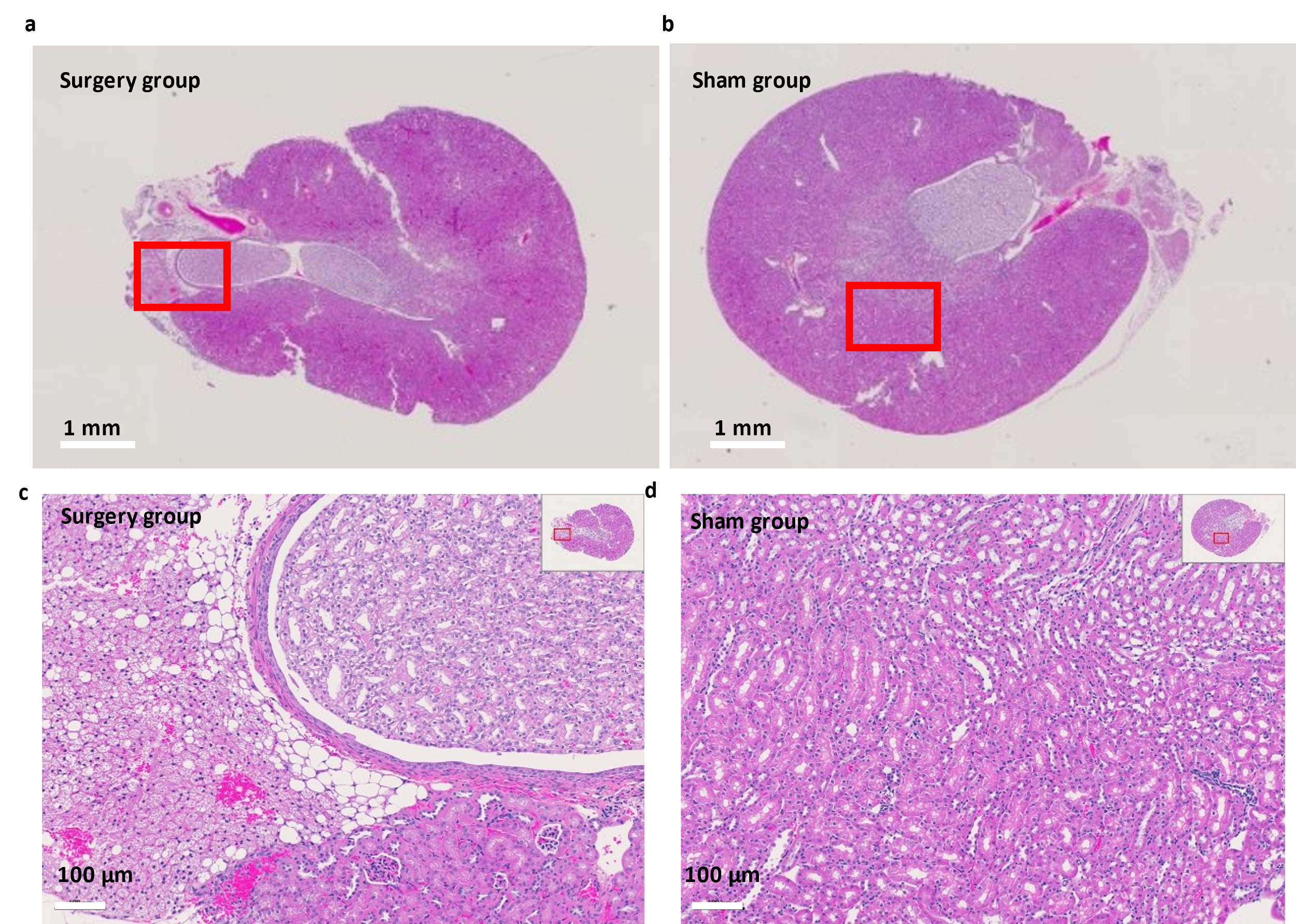


**Figure S15 (a)-(b) H&E stain images in surgery group and sham group. (c)-(d) Zoom-in H&E stain images in surgery group and sham group.**


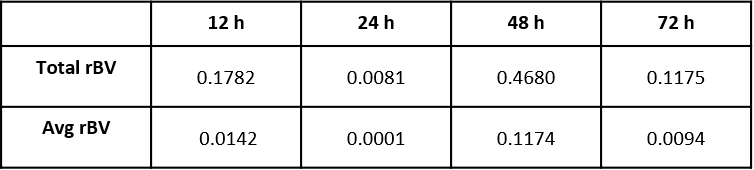

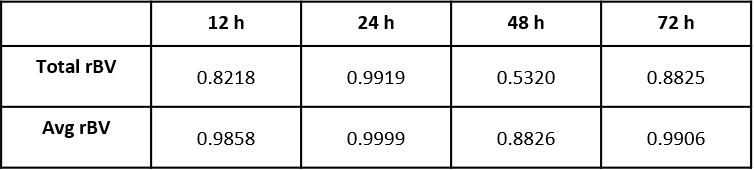


**Table S1: Statistical Power**

**Table S2: Type 2 error**
